# The association between family history and genomic burden with schizophrenia mortality: a Swedish population-based register and genetic sample study

**DOI:** 10.1038/s41398-021-01282-1

**Published:** 2021-03-15

**Authors:** Kaarina Kowalec, Yi Lu, Jie Song, Christina Dalman, Christina M. Hultman, Henrik Larsson, Paul Lichtenstein, Patrick F. Sullivan

**Affiliations:** 1 Department of Medical Epidemiology and Biostatistics, Karolinska Institutet, Stockholm, Sweden; 2 College of Pharmacy, University of Manitoba, Winnipeg, MN Canada; 3 Department of Global Public Health Sciences, Karolinska Institutet, Stockholm, Sweden; 4 Icahn School of Medicine, Department of Psychiatry, Mt Sinai Hospital, New York, NY USA; 5 School of Medical Sciences, Örebro University, Örebro, Sweden; 6 Departments of Genetics and Psychiatry, University of North Carolina at Chapel Hill, Chapel Hill, NC USA

**Keywords:** Personalized medicine, Schizophrenia

## Abstract

Individuals with schizophrenia (SCZ) have a 2–3-fold higher risk of mortality than the general population. Heritability of mortality in psychiatric disorders has been proposed; however, few have investigated SCZ family history and genetic variation, with all-cause and specific causes of death. We aimed to identify correlates of SCZ mortality using genetic epidemiological and genetic modelling in two samples: a Swedish national population sample and a genotyped subsample. In the Swedish national population sample followed from the first SCZ treatment contact until emigration, death or end of the follow-up, we investigated a standardised measure of SCZ family history. In a subgroup with comprehensive genetic data, we investigated the impact of common and rare genetic variation. Cox proportional hazards regression was used to estimate the association between various factors and mortality (all and specific causes). A total of 13727 SCZ cases fulfilled criteria for the population-based analyses (1268 deaths, 9.2%). The genomic subset contained 4991 cases (1353 deaths, 27.1%). Somatic mutations associated with clonal hematopoiesis with unknown drivers were associated with all-cause mortality (HR 1.77, 95% CI: 1.26–2.49). No other heritable measures were associated with all-cause mortality nor with any specific causes of death. Future studies in larger, comparable cohorts are warranted to further understand the association between hereditary measures and mortality in SCZ.

## Introduction

Individuals with schizophrenia (SCZ) have a 2 to 3-fold higher risk of mortality (mortality rate ratio = 2.57)^1^, resulting in 15–20 years shorter life expectancy, than the general population^2^. Reasons for the excess deaths in SCZ include high rates of poor health behaviours (e.g. smoking), high risk of suicides and accidents, adverse effects of antipsychotics and under-diagnosis/treatment of general medical comorbidities^3–5^. Approximately half of the excess deaths are due to natural or somatic causes^6,7^, particularly cardiovascular disease, lung cancer, chronic obstructive pulmonary disease and infections. Significant predictors of SCZ mortality include somatic and psychiatric comorbidities such as type 2 diabetes (odds ratio = 2.7)^8^, chronic obstructive pulmonary disease (odds ratio = 2.8)^8^, substance use disorder (hazard ratio = 2.0)^5^ or previous suicide attempts (hazard ratio = 2.3)^5^.

A heritable basis for mortality in psychiatric disorders has been proposed, including one study that found a family history of psychiatric admissions was associated with excess mortality in probands with a psychiatric disorder (mortality rate ratio = 1.1)^9^. Family history investigations of mortality specifically in those with SCZ have also shown an association, including a study reporting a significant effect between family history of mental disorders and SCZ mortality (mortality rate ratio = 1.8)^10^. However, there have been few studies of family history explicitly of SCZ and of specific causes of death. One study reported a lack of an association between SCZ family history and circulatory mortality^11^. Direct assessment of genetic variation can better capture genetic aspects of family history: a Danish study found no association between SCZ genetic risk score (GRS) and mortality^10^, but the number of deaths was small in this relatively young cohort and the impact of rare variation was not assessed^10^.

Given the impact of mortality in SCZ and the high heritability of SCZ^12^, we sought to comprehensively identify correlates of SCZ mortality using two samples. We conducted genetic epidemiological modelling of mortality in a Swedish national population sample of SCZ cases (13727 SCZ cases) and then investigated the impact of GRS of eight different traits or diseases, copy number variant measures, rare exome burden and somatic mutational burden in a genotyped subsample (4991 SCZ cases).

## Methods

### Study design and participants

The population-based analyses included SCZ cases born in Sweden between 1958–1994 (*n* = 13, 727) with register data available up to 2013. Participants were followed from the first SCZ treatment contact which occurred after January 1,1973 (the start of the Swedish National Patient Register) until emigration, death or the end of the available register data (December 31, 2013). The Regional Ethics Board of Stockholm approved the use of the anonymized population-based cohort. For genomic analyses, we used a subsample of 4991 SCZ cases from the Sweden SCZ Study^13^. The Regional Ethical Committee, Stockholm provided ethical approvals for the Sweden SCZ Study and all subjects provided written informed consent. Further details on the two samples are available in the Supplementary Material.

### Register data

Using Swedish personal identity numbers assigned at birth, we obtained linkages to multiple national registers. The National Patient Register provided admission dates and diagnoses for inpatient and specialist outpatient treatment^14^. The Cause of Death Register provided the date and causes of death^15^. The Multi-Generation Register allowed creation of pedigrees of first-degree relatives. Further details on the Swedish Register Data are available in the Supplementary Material.

For the population-based cohort, we generated a quantitative family history score for SCZ^16^. Briefly, we summarised the SCZ family history in parents, siblings or children for each case accounting for family size and age and sex distributions of the relatives. The SCZ family history score is the deviation from the expected family history in standard deviation units (Supplementary Material). Additional factors associated with SCZ mortality were considered and are described in the Supplementary Material (Supplementary Table 1).

### Genomic data

#### Common variant burden, genetic risk scores (GRS)

We included GRS of SCZ because those with a family history of psychiatric admission have a higher risk of mortality^9^. Cognitive ability GRS was included because lower cognitive ability is associated with SCZ mortality^17^. Additional GRS were selected because of associations between the traits or diseases and increased mortality from population samples: body mass index (BMI), coronary artery disease, total cholesterol, chronic kidney disease, type 2 diabetes and smoking. All training sets for computing GRS excluded Swedish samples.

#### Rare copy number variant (CNV) burden

CNV processing is described in detail elsewhere^18^, and summarised in the Supplementary Material. CNV burden is reported as the total burden for CNV size (in kilobases, kb) and total number of CNVs.

#### Rare exonic burden

Whole exome sequencing details are provided elsewhere^19^ and are summarised in the Supplementary Material. We computed the number of ultra-rare damaging and disruptive variants (ddURVs) in each case.

#### Clonal hematopoietic somatic mutations

Clonal hematopoiesis is the formation of genetically distinct blood cells from early progenitor cells, which play a role in proliferative diseases like haematologic cancers. Somatic mutations associated with this process are associated with increased mortality^20^. Whole exome sequencing was also used to identify somatic mutations associated with clonal hematopoiesis, as estimated from unusual allelic fractions. Further details of the genomic data processing are found in the Supplementary Material.

### Outcomes

For each death, we used the date of death and the underlying cause of death from the Swedish Cause of Death register^15^. Causes are coded using the World Health Organisation International Classification of Diseases (ICD, Supplementary Table 2) according to versions 8 (1973–1986), 9 (1987–1996) or 10 (1997 onwards).

### Statistical analysis

For the national population cohort, we described the SCZ cases who died compared to those who did not die during follow-up. We then generated unadjusted and adjusted Cox proportional hazards regression models to test the association between candidate factors and SCZ mortality (all-cause and specific causes), and were followed from the first treatment contact for SCZ until emigration, death or end of the follow-up period. The unadjusted model includes only the exposure of interest. The adjusted model included somatic and psychiatric conditions based on previously established associations with SCZ mortality or if they reached a pre-specified significance level in the unadjusted analysis. Further details of the covariates are found in the Supplementary Material.

For the subset with genetic data, we again used Cox proportional hazards regression models to test the association between genetic factors and SCZ mortality (all-cause and specific causes). Three separate models of inherited genetic factors were evaluated adjusting for the first five ancestry principal components: (1) common genetic burden scores evaluated eight different GRS; (2) rare CNV burden; and (3) rare exome burden included the count of ddURVs, and the count of synonymous variants (as a technical covariate).

Additionally for the genetic subset, we considered a separate Cox regression model including somatic mutations previously linked with clonal hematopoiesis^20^ in association with SCZ mortality. We modelled the effects of these somatic mutations as either those with candidate drivers of clonal hematopoiesis and separately for those with unknown drivers of clonal hematopoiesis. We derived unadjusted and adjusted Cox regression models for clonal hematopoiesis with somatic mutations in association with SCZ mortality (all-cause and specific causes). The adjusted model included age^20^, sex^21^ and the first five ancestry principal components^22^, given their associations with these somatic mutations.

All analyses were performed using *R* for statistical computing (version 3.6.1) and *R*-Studio (version 1.2.5001). Hazard ratios (HR) and 95% CI are reported. All statistical tests were two-sided. Due to the number of tests performed, we used a Bonferroni-adjusted *p* value of *p* ≤ 0.0011 indicating significance (0.05/45 tests = 7 models in the population-based analyses and 38 models in the genetic subsample).

## Results

Our goal was to identify hereditary factors associated with SCZ mortality using two cohorts. First, we used genetic epidemiological modelling of all-cause and specific causes of mortality in a Swedish national population sample (13,727 SCZ cases) and then investigated the impact of common and rare variant measures in a genotyped subsample (4991 SCZ cases).

For the population-based analyses, 13,727 SCZ cases born in Sweden between 1958–1994 were followed from the first treatment contact for SCZ until emigration, death or end of the follow-up period (December 31, 2013): 119 SCZ cases emigrated (0.9%), 1268 cases died (9.2%) and 12340 SCZ cases (89.9%) were followed until the end of the follow-up period. Individuals were followed for a median of 11.2 years. SCZ cases who died over the follow-up period were more likely to be male, younger age, lower educational attainment, fewer outpatient visit but more inpatient visits and have somatic comorbidity, compared to SCZ cases who did not die (Table 1).

**Table 1:** Descriptive characteristics of schizophrenia cases who did or did not die during the study period from the population-based cohort and genomic subset.

	Population-based cohort (*n* = 13727)		Genomics subset (*n* = 4991)	
Characteristic	Died 1268 (9.2%)	Did not die 12459 (90.8%)	Died 1353 (27.1%)	Did not die 3638 (72.9%)
Male sex	902, 71.1%	7537, 60.5%	841, 62.2%	2180, 59.9%
Age^a^, years	38 (30, 45)	45 (37, 51)	68 (60, 74)	62 (54, 69)
Number of SCZ outpatient visits	0 (0, 2.6)	3.3 (2, 4.2)	2.8 (1, 3.7)	3.8 (2.3, 4.7)
Number of SCZ inpatient hospitalisations	2.6 (2, 3.7)	2.0 (1, 3.3)	3.3 (2.3, 4.2)	3.2 (2.3, 4.1)
Any antipsychotic use	594, 46.9%	11790, 94.6%	1310, 96.8%	3438, 94.5%
Educational attainment^b^ (*Z*-score)	−0.40 (0.8)	−0.31 (0.9)	−0.44 (0.96)	−0.28 (0.86)
SCZ family history score^c^ (*Z*-score)	0.7 (2.4)	0.7 (2.6)	NA	NA
SCZ family history (binary)	125, 9.9%	1161, 9.3%	207, 15.3%	592, 16.3%
Suicide attempts	214, 16.9%	1955, 15.7%	132, 9.8%	471, 12.9%
Substance use disorder	477, 37.6%	3289, 26.4%	365, 26.9%	941, 25.9%
Major depressive disorder	257, 20.3%	3693, 29.6%	335, 24.8%	996, 27.4%
Diabetes	107, 8.4%	813, 6.5%	419, 30.9%	630, 17.3%
Chronic obstructive pulmonary disease	36, 2.8%	202, 1.6%	330, 24.4%	283, 7.8%

Categorical variables are presented as *n*, % and continuous are presented as the mean (standard deviation) for *Z*-score standardisation variables or median (interquartile range) for all other continuous variables.

*NA* not applicable.

^a^Age at emigration, death or end of follow-up period.

^b^For the population-based cohort, data available for 1112 died, 12263 did not die.

^c^For the population-based cohort, data available for 1179 died, 12096 did not die.

In the genomic subset, 4991 SCZ cases were included. By the end of the follow-up, 1353 SCZ cases died (27.1%). The genotyped subset was followed for a median of 9 years. This subset was an older cohort compared to the population-based sample, and hence had higher number of deaths and somatic comorbidities. The SCZ cases who died in the genomic subset were similar to those that died in the population-based cohort, with males, those with fewer outpatient visits, and lower education being more prevalent (Table 1).

### All-cause mortality

**Fig. 1:**
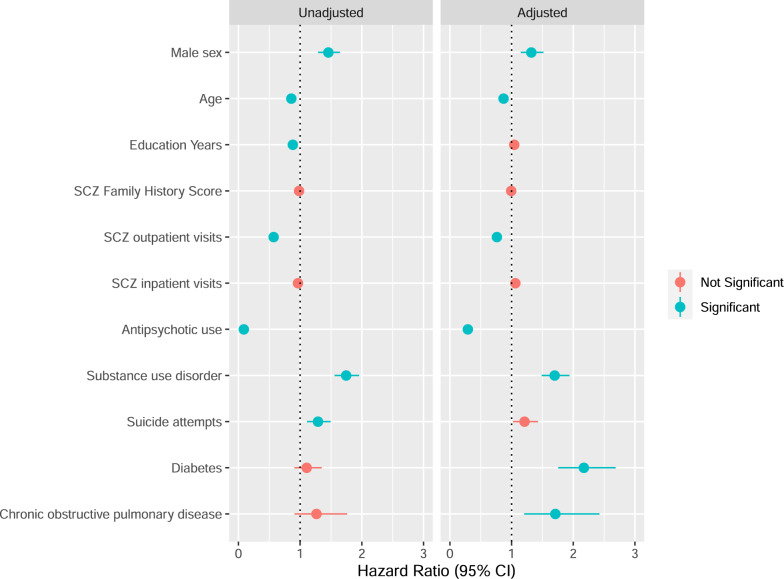
Cox proportional hazards regression modelling of factors associated with mortality in a Swedish national population-based sample of schizophrenia (SCZ) cases. The unadjusted model considers each factor individually and the adjusted model includes all listed factors in one model. The adjusted model additionally contains major depressive disorder. Total *N* for all exposures in the unadjusted model: 13,727 SCZ cases (1268 died), except for education years (13,375 SCZ cases, 1112 died) and SCZ family history score (13,275 SCZ cases, 1179 died). Total *N* for the adjusted model: 12,973 SCZ cases (1064 died). The information in this figure is the same as what is represented in Supplementary Table 3. NS not significant.

We investigated whether SCZ family history score was associated with all-cause mortality in SCZ. The median age at death in the population-based SCZ cohort was 38 years old (interquartile range: 30, 45). The multivariable Cox regression model for all-cause mortality in the population-based cohort included 12973 individuals (Fig. 1 and Supplementary Table 3). The following factors were significantly associated with an increased hazard of all-cause mortality in SCZ in the adjusted model: male sex, younger age, somatic conditions, fewer SCZ outpatient visits, no antipsychotic use and substance use disorder. SCZ family history score was not associated with SCZ mortality in the unadjusted or adjusted Cox regression models at our Bonferroni-adjusted *p* value (adjusted HR: 0.99, 95% CI: 0.97–1.02). Instead of inpatient SCZ admissions, we included the number of median SCZ bed days in the multivariable model, but the findings remained the same as previous, with SCZ family history score not associated with SCZ mortality (data not shown).

We performed analyses in the genomic subset to test the association of mortality with a comprehensive set of genomic burden measures. The median age at death in the genomic cohort was 69 years old (interquartile range: 62, 76). We assessed GRS of SCZ, four complex traits (cognitive ability, BMI, total cholesterol and smoking), and three somatic diseases (coronary artery disease, chronic kidney disease and type 2 diabetes) (Supplementary Table 4). No GRS reached our Bonferroni-adjusted significance threshold (*p* > 0.0011) in association with SCZ all-cause mortality (Supplementary Fig. 1) in the ancestry principal component adjusted model or the fully adjusted model. We also assessed the association between mortality and several rare variant CNV or exonic burden measures, and none reached the Bonferroni-adjusted significance threshold (Supplementary Fig. 1).

Although inherited genetic variation was not associated with all-cause mortality in SCZ, we also considered somatic mutations associated with clonal hematopoiesis (Supplementary Table 5). We report that SCZ cases with clonal hematopoiesis associated somatic mutations with unknown drivers were at a significantly increased hazard of all-cause mortality in SCZ after adjusting for age, sex and genetic ancestry (HR: 1.77, 95% CI: 1.26–2.49). There is a strong association between age and the presence of clonal hematopoiesis associated somatic mutations, with the most frequent class of these mutations occurring at CpG dinucleotides^23^. Thus, we also tested whether methylation age acceleration (a biological age predictor) was associated with these somatic mutations using a small subset of the genomic sample (*n* = 189 SCZ cases) which also had methylation data (Supplementary Material)^24^. However, there was no significant association between methylation age acceleration and the presence of these somatic mutations (*p* > 0.1, data not shown).

### Specific causes of mortality

We investigated whether specific causes of mortality were associated with SCZ family history score in the population-based cohort (Table 2). We had data on 1179 individuals on the underlying cause of death and SCZ family history (*n* = 1179/1268, 92.9%, Table 2) and were included in the analyses. The most common cause of death in the population-based sample was ‘external causes' (e.g. accidents or suicides, *n* = 431, 36.5%), followed by ‘other causes' (*n* = 260, 22.0%), cardiovascular disease (*n* = 187, 15.9%), and cancer (*n* = 76, 6.4%). The most common ‘other causes' of death included: other ill-defined and unspecified causes of mortality (*n* = 36), shock, unspecified (*n* = 22) and abnormal tumour markers (*n* = 16).

**Table 2:** Cox proportional hazards modelling of schizophrenia family history in association with specific causes of death in population-based sample of Swedish cases of schizophrenia (Population-based cohort, *n* = 1179 SCZ cases who died).

Outcome	Died from specific cause, *n*	Died from other cause, *n*	Schizophrenia family history score, *Z*-score^a^
Any cancer	76	1103	1.07 (0.98–1.16, *p* = 0.138)
Breast cancer	6	1173	1.18 (0.93–1.49, *p* = 0.183)
Lung cancer	8	1171	1.02 (0.76–1.39, *p* = 0.881)
Cardiovascular disease	187	992	1.08 (1.03–1.14, *p* = 0.003)
Stroke	17	1162	0.87 (0.61–1.24, *p* = 0.438)
External causes	431	748	0.98 (0.94–1.03, *p* = 0.422)
Suicide	423	756	0.98 (0.94–1.03, *p* = 0.449)
Other causes	260	919	1.01 (0.96–1.07, *p* = 0.647)

^a^Hazard ratio (95% confidence interval, *p* value) presented considers each outcome individually and the schizophrenia family history score. No effects were significant at the Bonferroni-adjusted *p* value.

SCZ family history score was not significantly associated with any causes of death in individuals with SCZ. Higher SCZ family history score increased the hazard of dying due to cardiovascular disease (HR: 1.08, 95% CI: 1.03–1.14, *p* = 0.003), although this finding did not surpass our stringent Bonferroni-adjusted significance threshold.

We then investigated whether common and rare genetic variant measures were associated with specific causes of death in the genomics sample (Table 3). The most common cause of death was ‘other causes' (*n* = 391, 31.2%), followed cardiovascular disease (*n* = 323, 25.7%) and cancer (*n* = 227, 18.1%). The most common ‘other causes' of death included: other ill-defined and unspecified causes of mortality (*n* = 29), chronic obstructive pulmonary disease with acute lower respiratory infection (*n* = 26) and unspecified dementia (*n* = 16).

**Table 3:** Cox proportional hazards modelling of common variant genetic risk scores in association with specific causes of death in Swedish cases of schizophrenia (Genomics subset, *n* = 1253 SCZ cases who died).

			Common variant genetic risk scores							
Cause of death	Died from specific cause, *n*	Died from other cause, *n*	SCZ	Cognitive ability	BMI	Coronary artery disease	Total cholesterol	Chronic kidney disease	Smoking	Type 2 diabetes
Any cancer	227	1026	1.08 (0.93–1.27)	0.79 (0.68–0.92, *P* = 0.002)	0.94 (0.81–1.08)	0.99 (0.87–1.14)	1.01 (0.88–1.15)	1.13 (0.98–1.29)	0.86 (0.75–0.98)	0.94 (0.81–1.09)
Breast cancer	11	1242	1.59 (0.74–3.44)	0.65 (0.31–1.40)	0.78 (0.38–1.60)	1.24 (0.62–2.50)	0.54 (0.27–1.07)	1.01 (0.51–1.99)	0.72 (0.37–1.43)	0.70 (0.32–1.50)
Lung cancer	66	1187	0.97 (0.72–1.31)	0.87 (0.66–1.16)	1.08 (0.83–1.40)	0.96 (0.75–1.23)	1.05 (0.82–1.34)	0.99 (0.77–1.28)	0.71 (0.55–0.91)	0.91 (0.69–1.19)
Cardiovascular disease	323	930	0.99 (0.87–1.12)	0.94 (0.83–1.06)	0.94 (0.84–1.06)	0.95 (0.84–1.06)	0.98 (0.88–1.09)	0.99 (0.88–1.11)	0.97 (0.87–1.09)	1.08 (0.95–1.22)
Stroke	60	1193	0.88 (0.65–1.20)	1.04 (0.78–1.39)	0.95 (0.72–1.25)	0.94 (0.72–1.24)	0.91 (0.70–1.18)	0.94 (0.72–1.23)	1.20 (0.92–1.55)	0.97 (0.73–1.28)
External causes	75	1178	1.17 (0.89–1.54)	1.00 (0.77–1.29)	1.12 (0.88–1.44)	0.96 (0.76–1.22)	1.08 (0.86–1.35)	1.06 (0.84–1.35)	0.98 (0.78–1.24)	0.97 (0.75–1.25)
Suicide	31	1222	0.86 (0.56–1.31)	0.85 (0.57–1.27)	0.90 (0.62–1.32)	0.90 (0.61–1.32)	0.66 (0.46–0.94)	0.89 (0.62–1.29)	0.87 (0.60–1.25)	0.72 (0.49–1.06)
Other causes	391	862	1.01 (0.89–1.14)	1.07 (0.95–1.20)	1.01 (0.91–1.13)	1.02 (0.92–1.13)	1.05 (0.95–1.16)	0.94 (0.84–1.04)	1.01 (0.91–1.12)	0.97 (0.87–1.08)

Hazard ratios (95% confidence interval) are presented. No effects statistically significant at the Bonferroni-adjusted *p* value. All exposures (genetic risk scores) are included in a single model along with the first five ancestry principal components.

We assessed the association between eight common variant GRS and specific causes of death in SCZ (Table 3). We found that SCZ GRS was not significantly associated with any specific cause of death. No other GRS were significantly associated with specific causes of death, however, higher cognitive ability GRS decreased the hazard of dying due to cancer (HR: 0.79, 95% CI: 0.68–0.92, *p* = 0.002), although this finding did not surpass the Bonferroni-adjusted significance threshold. We assessed whether rare genetic burden measures for CNVs and ddURVs were associated with dying from specific causes of death but none reached significance (Supplementary Table 6). For somatic mutations associated with clonal hematopoiesis, neither the unadjusted nor the adjusted regression analyses demonstrated a significant effect of these mutations on specific causes of death in SCZ cases (Supplementary Table 7).

## Discussion

We conducted a comprehensive assessment of SCZ mortality using population-based register and genomic data. We used two Swedish SCZ samples—a national population sample (13727 cases) and a genotyped subsample (4991 cases)—to provide a better understanding of the correlates of SCZ mortality. We found that all-cause mortality and specific causes of death in Swedish SCZ cases were unrelated to a standardised measure of SCZ family history and to a broad set of germline genetic factors (i.e. inherited genetic mutations: eight common variant GRS, CNVs and rare exome measures).

Somatic mutations associated with clonal hematopoiesis were associated with all-cause mortality in SCZ. We assessed two measures of clonal hematopoiesis: with and without candidate driver mutations. We found that those with evidence of clonal hematopoiesis and unknown drivers were at ~75% increased hazard of all-cause mortality in SCZ (adjusting for age, sex and genetic ancestry). Overall, the presence of clonal hematopoiesis of indeterminate potential (CHIP) increases markedly with age^25^. CHIP was originally associated with risk of hematopoietic cancer^20,26,27^ but now appears to be a potent risk factor for all-cause mortality and multiple forms of cardiovascular disease^27^. The magnitude of risk may exceed established risks for cardiovascular disease (e.g. smoking or hypertension). We provide an update to an earlier report of SCZ cases and controls from this study^20^ by adding more than 5 years of follow-up from the Swedish Cause of Death register, and found a robust association with all-cause mortality specifically in SCZ cases. Reduced survival was not explained by any specific cause of death (*p* > 0.05) but with the caveat that the numbers per specific cause of death category were small (<20). Our findings are consistent with CHIP as a risk factor for mortality of potential clinical relevance.

Notably, none of the inherited measures (SCZ family history, multiple common GRS, rare CNV and rare exonic mutation) were associated with all-cause or specific causes of mortality in SCZ. This could be due to the heterogeneity captured by all-cause mortality and provides additional rationale for investigating more homogeneous outcomes, such as specific causes of mortality in larger cohorts. This is also notable given that we significantly increased the number of SCZ cases that died in comparison to a previous genetic study of SCZ mortality^10^. The lack of any significant findings in the current study could be due to either different or unmeasured genetic or environmental factors that play a role or that a larger sample size is needed. Of note, we had two findings that did not pass our Bonferroni-adjusted significance threshold, but could represent future areas for investigation into SCZ mortality. One, we found that SCZ family history score was linked with death due to cardiovascular disease and could be due to shared genetic and/or environmental influences between cardiovascular disease and SCZ, as they are known to share pathological mechanisms^28,29^. Second, we found the common variant burden measure of cognitive ability (IQ GRS) was linked with a decreased hazard of cancer death. Studies investigating the genetic determinants of IQ in association with mortality are lacking in the general population and in SCZ. However, lower IQ scores have been associated with increased mortality risk in the general population^30^ and in SCZ^17^. In addition, it is worthwhile to note that intelligence testing measures multiple domains of cognitive functioning and is subject to environmental influences, whereas genetic determinants of cognitive ability do not change from conception. Here, we present a unique opportunity to guide future research into the determinants of specific causes of mortality given our finding of genetic determinants of cognitive ability associated with cancer deaths in SCZ.

The strengths of our study include large samples of SCZ cases with both comprehensive register and genomic data from a single country. To the best of our knowledge, specific causes of death in association with various genomic and register have not yet been investigated within a SCZ population. We also included a comprehensive set of genomic factors, including somatic mutations associated with clonal hematopoiesis. Limitations of this study included different age profiles of the two samples. A higher overall number of deaths in the genomics subset occurred because of the older age of SCZ cases relative to the population-based cohort. The number of deaths in each cause of death category differed between the two samples, with more deaths due to external causes in the population-based sample. The rate of suicide deaths in the population-based sample was higher compared to epidemiological studies^31,32^, although it should be noted that the purpose of this study was to investigate associations between hereditary measures and mortality, not to define rates of death due to specific causes. The genetic subsample was an older sample, hence more deaths from somatic conditions occurred and fewer by external causes, such as suicides. Other studies have identified a genetic transmission of suicide^33^, however, only 31 deaths due to suicide were recorded in our genetic sample. We were also limited by the timelines of the available register data. For example, the Swedish National Patient Register began including psychiatric diagnoses in 1973, had 50% of the Swedish population included by 1976 and attained complete coverage of the Swedish population by 1987^14^. By including individuals born in 1958, we likely underestimated the number of SCZ cases. Lastly, further investigation of the heritable component of SCZ mortality in larger cohorts, possibly in neighbouring Nordic countries with similar register data, with a larger sample of deaths are indeed warranted.

In conclusion, we investigated a broad range of register and genomic factors in association with all-cause and specific causes of SCZ mortality in two Swedish samples. We found that all-cause mortality in SCZ was related to CHIP but unrelated to familial and other genetic burden measures. Specific causes of death were not associated with heritable measures. Future studies in SCZ, incorporating a larger sample of deaths with comprehensive genetic measures, could further elucidate the possible mechanisms underlying specific causes of death.

## Supplementary information

Supplementary material

Figure S1

## Data Availability

Custom *R* scripts used for the statistical analyses in this study can be provided upon reasonable request to the authors.
