# Supplementary material for: The association between family history and genomic burden with schizophrenia mortality: a Swedish population-based register and genetic sample study
Source: Transl Psychiatry. 2021 Mar 15;11:163. doi: 10.1038/s41398-021-01282-1 (PMC7960991; doi:10.1038/s41398-021-01282-1)
Supplement: Supplementary file 1 — Supplementary material [file 41398_2021_1282_MOESM1_ESM.docx]

#### **Supplementary Information**

#### **Supplementary methods**

#### **Study design and population details**

For the population-based cohort study, the source population for SCZ cases was defined as those who were hospitalized ≥2 times with a SCZ or a schizoaffective disorder discharge diagnosis (***Supplementary*** ***Table 1*** includes the International Classification of Diseases [ICD] codes) after the start of the Sweden National Patient Register^1–3^ (January 1, 1973, N=48,555). The criteria were born in Sweden between 1958-1994 and exclude those with high treatment contacts for substance use disorder (≥50 substance use disorder treatment contacts and ≤5 SCZ treatment contacts) or bipolar disorder (≥5 bipolar treatment contacts and ≤5 SCZ treatment contacts) (n=13,727). Participants were followed from this baseline (January 1, 1973) until whichever came first: emigration, death, or end of the register data period (December 31, 2013). Somatic and psychiatric conditions were identified using the ICD codes listed in ***Supplementary*** ***Table 1*** as recorded in the National Patient Register.^2,3^ The ethical approval permitted the investigation of the included ICD codes for somatic conditions only as comorbidities. Other factors considered in the population-based analyses included factors previously associated with SCZ mortality: age, sex, antipsychotic drug prescriptions,^4^ type 2 diabetes,^5^ chronic obstructive pulmonary disease,^5^ substance use disorder,^6^ or previous suicide attempts.^6^

The family history score (FH_i_) of SCZ in the population-based analyses was generated using first-degree relatives (parents, siblings, children) of the *i*th proband and the following:

$${FH}_{i}=\sum_{j} \frac{O_{i,j}-E_{i.j}}{\sqrt{E_{i,j}}}$$

In the equation, *O* codes for whether the *j*th relative of the *i*th proband had SCZ and *E* is the expected lifetime SCZ prevalence (given the age and sex per proband and relative). The values are summed over *j* relatives of the *i*th proband. The expected lifetime prevalence of SCZ were generated using all individuals in Sweden. In the genomic subset, family history for SCZ was dichotomized as negative (no first-degree relative with SCZ) or positive (at least one-first degree relative with SCZ).

For the genetic analyses, we used a sample of the Sweden Schizophrenia Study (S3),^7^ a cohort of 4,991 Swedish SCZ cases recruited between 2004-2010 and had register data up to December 31, 2018. Subjects were 18 years of age or older. The average age at sampling was 53 years. Cases were identified from the Swedish National Patient Register,^2,3^ using the same definition as the population-based analyses: hospitalized ≥2 times with a SCZ or schizoaffective disorder discharge diagnosis (***Supplementary*** ***Table 1***) and excluded those with high treatment contacts for substance use disorder (≥50 substance use disorder treatment contacts and ≤5 SCZ treatment contacts) or bipolar disorder (≥5 bipolar treatment contacts and ≤5 SCZ treatment contacts).^8^ The SCZ definition has been validated clinically, genetic epidemiologically, and genetically,^9^ and has high agreement with medical^2,3^ and psychiatric diagnoses.^10^ Participants were followed from DNA sampling until death or end of the register data period (December 31, 2018). Emigration data was unavailable for this cohort.

Age in both cohorts was defined as age at emigration (for population-based only), death, or end of the follow-up period. The number of SCZ in- and outpatient admissions were log-transformed.

#### **Data sources**

Years of education were provided by the Longitudinal Integration Database for Health Insurance and Labour Market.^11^ For the population-based cohort, the Migration Register contained emigration dates throughout the study period. The Swedish National Prescribed Drug Register provided redeemed prescriptions for antipsychotics (Anatomical Therapeutic Chemical [ATC] Classification: N05A) in outpatient care.^12^

#### **Genomic data processing**

#### **Genetic risk scores (GRS), common variant burden**

Genotype data of the target dataset were processed using the PGC Ricopili pipeline.^13^ We excluded samples for missingness or high relatedness ($\hat{\pi}$ ≥0.2). We excluded variants if they were not biallelic, lacked a dbSNP rsID, were strand ambiguous, had an allele frequency <0.05 or >0.95, or poor imputation (INFO score <0.8).^14^ We applied linkage disequilibrium-based clumping to select an independent set of single nucleotide polymorphisms (SNPs) to calculate GRS (r^2^ < 0.1 in 1 Mb windows using 1000 Genomes Project European samples as reference). Using PLINK (v1.9),^15^ GRS were calculated in the target dataset as the sum of the SNP dosages weighted by the effect from the discovery summary statistics set across all SNPs under the pre-specified *P*-value threshold (*p*≤0.05). We obtained GWAS results for SCZ^9^ , IQ,^16^ BMI,^17^ coronary artery disease,^18^ total cholesterol,^19^ chronic kidney disease,^20^ type 2 diabetes,^21^ and smoking (cigarettes per day).^22^ GRS were selected either because of associations with mortality in SCZ^23,24^ or in population samples: body mass index (BMI),^25^ coronary artery disease,^18^ total cholesterol,^19^ chronic kidney disease,^20^ type 2 diabetes,^21^ and smoking.^22^ There were four genotyping waves in our target dataset and thus GRS were standardised to each wave to account for variation in the numbers of SNPs used across different genotyping chips. We removed a small number of outliers, leaving 4,681 SCZ cases with GRS computed.

#### **Rare copy number variant (CNV) burden**

CNV processing details are provided previously,^26^ but briefly, CNVs were detected using Birdseye.^27^ Quality control included removing low-confidence CNVs (confidence scores <10, spanning <10 probes, or <10 kb in length) followed by removal of CNVs with >50% reciprocal overlap with large genomic gaps (e.g., centromeres) or regions subject to rearrangement in white blood cells. Adjoining CNVs were annealed that appeared to be artificially split by Birdseye by recursively joining CNVs, if the called region was ≥80% of the entire region to be joined. We visually inspected the largest CNVs (≥5 Mb) and chrX CNVs and removed those of low confidence. Subjects were excluded if they had excessive noise or CNV calls scattered across many chromosomes (≥40 segments or total length ≥ 6 Mb). We removed a small number of outliers, leaving 4,372 SCZ cases with CNV data. CNV data were then log-transformed and are presented as the total number of CNVs and the total size of all CNVs.

#### **Whole exome sequencing**

Whole exome sequencing details have been reported^28,29^ but, briefly, sequencing was performed using Agilent SureSelect Human All Exon kits and sequenced on Illumina GAII, HiSeq 2000, or HiSeq 2500 instruments (76 bp paired-end sequencing). Sequencing reads were aligned to the GRCh37 human genome reference and processed using GATK,^30^ with calls generated using GATK Haplotype Caller. We excluded variants filtered out by the GATK Variant Quality Score Recalibration tool and any sites with <10x coverage were set to missing. We removed a small number of outliers, leaving 4,681 SCZ cases with whole exome data.^28^ We computed the number of ultra-rare damaging and disruptive variants (ddURVs) not observed in the Exome Aggregation Consortium,^31^ and then values were log-transformed.

#### **Somatic mutations associated with clonal hematopoiesis**

We identified SCZ cases with somatic mutations associated with clonal hematopoiesis with either candidate or unknown drivers.^32^ Somatic mutations without a candidate driver were defined as alleles with the following criteria: a SNP, observed once or twice (MAF <0.01%) in the cohort, an allelic fraction >10%, and did not have a binomial distributed alternate allelic count (with mean 45% with a designed false positive rate of 10^-5^). Somatic mutations with a candidate driver were defined as those alleles satisfying the following criteria: SNPs or indels of length one or two base pairs, observed ≤6 times (MAF <0.025%) in the cohort, an allelic fraction >5%, and did not have a binomial distributed alternate allelic count (mean=47% for SNPs and 40% for indels with a false positive rate of 0.01). In addition, alleles with candidate driver somatic mutations satisfied the following: disruptive and missense mutations in the *DNTMT3A* gene (in exons 7 to 23), disruptive mutations in gene *ASXL1* (excluding *ASXL1* p.G646fsX12 and p.G645fsX583), disruptive mutations in gene *TET2,* disruptive mutations in gene *PPM1D,* missense mutation *JAK2* p.V617F, or mutations reported ≥ 7 times in hematopoietic and lymphoid malignancies using the Catalogue of Somatic Mutations in Cancer^33^ (and excluding inherited mutations and potential PCR artifacts). Select somatic mutations were also validated using targeted sequencing.

#### **Methylation age estimation**

Given the strong association between age and the presence of clonal hematopoiesis associated somatic mutations, and since the most frequent class of these mutations occurs at CpG dinucleotides, we tested whether methylation age acceleration (a biological age predictor) was associated with these somatic mutations. The methylation data details have been reported.^34^ Briefly, DNA was extracted from blood samples and DNA methylation was quantified using the Illumina Infinium HumanMethylationEPIC BeadChip (866,562 methylation sites across the genome) (Illumina Inc, CA, USA). Methylation probes were removed if >1% of samples have detection *p*-value <0.05 or if >5% of samples had a bead count <3. The methylation data were then quantile-normalized using the dasen function from the *wateRmelon* R package.^35^ One sample whose predicted sex did not match their reported sex was excluded during quality control, leaving a total of 189 SCZ samples and 809,996 methylation probes for analysis. Methylation age was computed using the Hannum algorithm, which is best suited for blood samples,^36^ and is based on 71 methylation probes. We also computed a smoking score (continuous measure) based on DNA methylation sites known to be associated with smoking^37^ and to account for methylation differences between cell types, we estimated the cell-type composition of the blood samples using *minfi* R package.^38^ The residuals resulting from a linear regression of methylation age on age at sampling were used as the measure of methylation age acceleration.

#### **Statistical analyses**

Categorical variables are presented as n, % and continuous are presented as the mean (standard deviation) for Z-score standardization variables or median (interquartile range) for all other continuous variables. All statistical models included cases with complete data on all exposures and outcomes. Specifically, for the subset of SCZ cases with methylation data (n=189), we tested the association between methylation age acceleration and the presence of somatic mutations using a Cox regression model including blood cell counts and smoking scores as covariates.

**Supplementary References**

1 Ludvigsson JF *et al.* External review and validation of the Swedish national inpatient register. *BMC Public Health* 2011; **11**: 450.

2 Kristjansson E, Allebeck P, Wistedt B. Validity of the diagnosis schizophrenia in a psychiatric inpatient register: A retrospective application of DSM-III criteria on ICD-8 diagnoses in Stockholm county. *Nord Psykiatr Tidsskr* 1987; **41**: 229–234.

3 Dalman C, Broms J, Cullberg J, Allebeck P. Young cases of schizophrenia identified in a national inpatient register. *Soc Psychiatry Psychiatr Epidemiol* 2002; **37**: 527–531.

4 Tiihonen J *et al.* 11-year follow-up of mortality in patients with schizophrenia: a population-based cohort study (FIN11 study). *Lancet* 2009; **374**: 620–627.

5 Schoepf D, Uppal H, Potluri R, Heun R. Physical comorbidity and its relevance on mortality in schizophrenia: A naturalistic 12-year follow-up in general hospital admissions. *Eur Arch Psychiatry Clin Neurosci* 2014; **264**: 3–28.

6 Limosin F, Loze JY, Philippe A, Casadebaig F, Rouillon F. Ten-year prospective follow-up study of the mortality by suicide in schizophrenic patients. *Schizophr Res* 2007; **94**: 23–28.

7 Ripke S *et al.* Genome-wide association analysis identifies 13 new risk loci for schizophrenia. *Nat Genet* 2013; **45**: 1150–9.

8 Lichtenstein P *et al.* Recurrence risks for schizophrenia in a Swedish National Cohort. *Psychol Med* 2006; **36**: 1417–1425.

9 Schizophrenia Working Group of the Psychiatric Genomics Consortium. Biological insights from 108 schizophrenia-associated genetic loci. *Nature* 2014; **511**: 421–7.

10 Ekholm B *et al.* Evaluation of diagnostic procedures in Swedish patients with schizophrenia and related psychoses. *Nord J Psychiatry* 2005; **59**: 457–464.

11 Statistics Sweden. Evaluation of the Swedish register of education. http://www.scb.se/statistik /_publikationer/BE9999_2006A01_BR _BE96ST0604.pdf (accessed 27 Mar2019).

12 Wettermark B *et al.* The new Swedish Prescribed Drug Register Opportunities for pharmacoepidemiological research and experience from the first six months. *Pharmacoepidemiol Drug Saf* 2007; **16**: 726–735.

13 Ripke S. Ricopili - Rapid Imputation Consortium Pipeline. https://sites.google.com/a/broadinstitute.org/ricopili/home (accessed 14 Aug2017).

14 Howie BN, Donnelly P, Marchini J. A flexible and accurate genotype imputation method for the next generation of genome-wide association studies. *PLoS Genet* 2009; **5**: e1000529.

15 Chang CC *et al.* Second-generation PLINK: Rising to the challenge of larger and richer datasets. *Gigascience* 2015; **4**: 7.

16 Savage JE *et al.* Genome-wide association meta-analysis in 269,867 individuals identifies new genetic and functional links to intelligence. *Nat Genet* 2018; **50**: 912–919.

17 Yengo L *et al.* Meta-analysis of genome-wide association studies for height and body mass index in ∼700000 individuals of European ancestry. *Hum Mol Genet* 2018; **27**: 3641–3649.

18 Nelson CP *et al.* Association analyses based on false discovery rate implicate new loci for coronary artery disease. *Nat Genet* 2017; **49**: 1385–1391.

19 Willer CJ *et al.* Discovery and refinement of loci associated with lipid levels. *Nat Genet* 2013; **45**: 1274–1285.

20 Wuttke M *et al.* A catalog of genetic loci associated with kidney function from analyses of a million individuals. *Nat Genet* 2019; **51**: 957–972.

21 Scott RA *et al.* An Expanded Genome-Wide Association Study of Type 2 Diabetes in Europeans. *Diabetes* 2017; **66**: 2888–2902.

22 Liu M *et al.* Association studies of up to 1.2 million individuals yield new insights into the genetic etiology of tobacco and alcohol use. Nat. Genet. 2019; **51**: 237–244.

23 Laursen TM, Munk-Olsen T, Nordentoft M, Mortensen PB. Increased mortality among patients admitted with major psychiatric disorders: A register-based study comparing mortality in unipolar depressive disorder, bipolar affective disorder, schizoaffective disorder, and schizophrenia. *J Clin Psychiatry* 2007; **68**: 899–907.

24 Dickerson F *et al.* Mortality in Schizophrenia: Clinical and Serological Predictors. *Schizophr Bull* 2014; **40**: 796–803.

25 De Gonzalez AB *et al.* Body-mass index and mortality among 1.46 million white adults. *N Engl J Med* 2010; **363**: 2211–2219.

26 Szatkiewicz JP *et al.* Copy number variation in schizophrenia in Sweden. *Mol Psychiatry* 2014; **19**: 762–773.

27 Korn JM *et al.* Integrated genotype calling and association analysis of SNPs, common copy number polymorphisms and rare CNVs. *Nat Genet 2008 4010* 2008; **40**: 1253.

28 Genovese G *et al.* Increased burden of ultra-rare protein-altering variants among 4,877 individuals with schizophrenia. *Nat Neurosci* 2016; **19**: 1433–1441.

29 Purcell SM *et al.* A polygenic burden of rare disruptive mutations in schizophrenia. *Nature* 2014; **506**: 185–190.

30 Poplin R *et al.* Scaling accurate genetic variant discovery to tens of thousands of samples. *bioRxiv* 2017; : 201178.

31 Lek M *et al.* Analysis of protein-coding genetic variation in 60,706 humans. *Nature* 2016; **536**: 285–291.

32 Genovese G *et al.* Clonal hematopoiesis and blood-cancer risk inferred from blood DNA sequence. *N Engl J Med* 2014; **371**: 2477–2487.

33 Forbes SA *et al.* COSMIC: mining complete cancer genomes in the Catalogue of Somatic Mutations in Cancer. *Nucleic Acids Res* 2011; **39**: D945-50.

34 Kowalec K *et al.* Methylation age acceleration does not predict mortality in schizophrenia. *Transl Psychiatry* 2019; **9**: 157.

35 Pidsley R *et al.* A data-driven approach to preprocessing Illumina 450K methylation array data. *BMC Genomics* 2013; **14**: 293.

36 Hannum G *et al.* Genome-wide Methylation Profiles Reveal Quantitative Views of Human Aging Rates. *Mol Cell* 2013; **49**: 359–367.

37 Elliott HR *et al.* Differences in smoking associated DNA methylation patterns in South Asians and Europeans. *Clin Epigenetics* 2014; **6**: 4.

38 Fortin JP, Triche TJ, Hansen KD. Preprocessing, normalization and integration of the Illumina HumanMethylationEPIC array with minfi. *Bioinformatics* 2017; **33**: 558–560.

39 World Health Organization. International Statistical Classification of Diseases and Related Health Problems, 10th Ed. 2010.http://www.who.int/classifications/icd/ICD10Volume2_en_2010.pdf (accessed 18 Aug2017).

40 Brooke HL *et al.* The Swedish cause of death register. *Eur J Epidemiol* 2017; **32**: 765–773.

41 Mollazadegan K *et al.* A population-based study of the risk of diabetic retinopathy in patients with type 1 diabetes and celiac disease. *Diabetes Care* 2013; **36**: 316–321.

42 Deleskog A *et al.* Severity of depression, anxious distress and the risk of type 2 diabetes - A population-based cohort study in Sweden. *BMC Public Health* 2019; **19**. doi:10.1186/s12889-019-7322-z.

43 Inghammar M, Engström G, Löfdahl CG, Egesten A. Validation of a COPD diagnosis from the Swedish Inpatient Registry. *Scand J Public Health* 2012; **40**: 773–776.

#### **Supplementary figure captions**

#### **Supplementary Figure 1:** Cox proportional hazards regression modelling of genomic factors associated with all-cause mortality in Swedish cases of schizophrenia (SCZ) (Genomics subset, N=4,991 SCZ cases). No factors were statistically significantly associated with SCZ mortality. The ancestry principal components adjusted model considers each genomic factor individually and the first 5 ancestry principal components. Three separate models are presented in the full adjusted model: 1) Eight genetic risk scores (GRS) and the first five principal components, 2) Two copy number variant (CNV) measures and the first 5 principal components, and 3) ultra-rare variant (URVs) burden measure, the first principal components and the count of synonymous variants as a technical covariate.

#### **Supplementary Table 1:** Classification of diseases and disorders according to the WHO international classification of diseases (ICD) version 8, 9 and 10 utilised in Sweden

|  | **ICD-8 and ICD-9** | **ICD-10** |
| --- | --- | --- |
| Schizophrenia^2,3,9,10^ | 295.0-295.4, 295.6-295.9 | F20, F20.0-F20.6, F20.8, F20.9 |
| Schizoaffective disorders^2,3,9,10^ | 295.7 | F25, F25.0-F25.2, F25.8, F25.9, F23.1, F23.2 |
| Major depressive disorder^39^ | ICD-8: 300.4  ICD-9: 296.3, 311 | F32, F32.0-F32.3, F32.8, F32.9, F33, F33.0-F33.4, F33.8, F33.9, F34.8, F34.9, F38, F38.0, F38.1-F38.8, F39 |
| Suicide^40^ | ICD-8: E950-E959  ICD-9: E95A-E95H, E95W, E95X | X60-X84 |
| Substance use disorder^39^ | ICD-8: 303, 303.1, 303.2, 303.9, 304, 304.1-304.9  ICD-9: 303, 303A, 303X, 304, 304A-304H, 304W, 304X, 305A, 305X | F10, F10.0-F10.9, F11, F11.0-F11.9, F19, F19.0-F19.9 |
| Diabetes^41,42^ | 250 | E10, E11 |
| Chronic Obstructive Pulmonary Disease^43^ | 491-492, 496 | J41–J44 |

#### **Supplementary Table 2:** Classification of causes of death according to the WHO international classification of diseases (ICD) version 8, 9 and 10 utilised in Sweden^40^

| **Category** | **ICD-8 and ICD-9** | **ICD-10** |
| --- | --- | --- |
| Alcohol | 305, 291, 303 | F10 |
| Suicide | E950-E959 | X60-X84 |
| Vehicle and other accidents | E800-E849 E850-E869, E880-E929 | V01-V99 W00-X59 |
| Cardiovascular disease | 390-429, 440-459 | I00-I59, I70-I99 |
| Stroke | 430-438 | I60-I69 |
| Prostate/breast cancer | ICD-8: 185 (prostate), 174 (breast)  ICD-9: 185 (prostate), 174-175 (breast) | Prostate: C61  Breast: C50 |
| Lung cancer | ICD-8: 162.1  ICD-9: 162.2-162.9 | C34 |
| Other cancers | ICD-8: 140-161, 163-173, 176-184, 186-239  ICD-9: 140-162.1, 163-173, 176-184, 186-239 | C00-C33, C35-C49, C51-C60, C62-C97 |
| Other | All remaining codes | |

**Supplementary Table 3:** Multivariable Cox regression analyses of factors associated with mortality in schizophrenia in a Swedish National population-based sample

|  | ***Unadjusted*** | | | ***Adjusted*** | | |
| --- | --- | --- | --- | --- | --- | --- |
| ***Characteristic*** | ***Hazard Ratio*** | ***Lower 95% bound*** | ***Upper 95% bound*** | ***Hazard Ratio*** | ***Lower 95% bound*** | ***Upper 95% bound*** |
| Male sex | **1.46** | **1.29** | **1.65** | **1.32** | **1.15** | **1.52** |
| Age | **0.85** | **0.85** | **0.86** | **0.87** | **0.86** | **0.88** |
| Education years, z-score | **0.88** | **0.82** | **0.94** | 1.04 | 0.97 | 1.12 |
| SCZ family history score, z-score | 0.98 | 0.96 | 1.01 | 0.99 | 0.97 | 1.02 |
| Number of SCZ outpatient visits | **0.57** | **0.55** | **0.59** | **0.76** | **0.73** | **0.80** |
| Number of SCZ inpatient visits | 0.97 | 0.93 | 1.00 | 1.06 | 1.01 | 1.11 |
| Any antipsychotic use | **0.08** | **0.08** | **0.10** | **0.29** | **0.25** | **0.34** |
| Substance use disorder | **1.75** | **1.56** | **1.96** | **1.70** | **1.49** | **1.94** |
| Suicide attempts | **1.29** | **1.11** | **1.49** | 1.21 | 1.03 | 1.43 |
| Major depressive disorder | 0.83 | 0.72 | 0.95 | 0.83 | 0.72 | 0.98 |
| Diabetes | 1.11 | 0.91 | 1.35 | **2.17** | **1.76** | **2.69** |
| Chronic obstructive pulmonary disease | 1.27 | 0.91 | 1.76 | 1.71 | 1.21 | 2.43 |

The information in this table is the same as what is represented in Figure 1. Bolded indicates statistically significant at *p*≤0.0011. Total N for all exposures in the unadjusted model: 13,727 SCZ cases (1,268 died), except for education years (13,375 SCZ cases, 1,112 died) and SCZ family history score (13,275 SCZ cases, 1,179 died). Total N for the adjusted model: 12,973 SCZ cases (1,064 died). The adjusted model includes all factors listed.

#### **Supplementary Table 4:** Genomic descriptive characteristics of schizophrenia cases who did or did not die during the study period from the genomic subset (n=4,991 SCZ cases).

| Characteristic | **Died** | **Did not die** |
| --- | --- | --- |
| Genetic scores, standardised^1^ (N=1,253/3,428)SCZCognitive ability Body mass index Coronary artery disease Total cholesterol  Chronic kidney disease  Smoking  Type 2 diabetes | 0.37 (0.90) -0.11 (1.01)  -0.02 (0.99)  -0.04 (1.03)  0 (1.00)  0.01 (1.00)  -0.06 (1.01)  0.04 (1.01) | 0.43 (0.95) -0.12 (1.04)  -0.04 (1.01)  -0.06 (1.05)  0 (1.00)  0.04 (1.05)  -0.01 (1.02)  0.07 (1.05) |
| Copy number variant (CNV) burden (N=1,172/3,200)Number of CNVsSize of CNV (kb) | 1 (0, 1) 7.04 (0, 8.6) | 1 (0, 1) 7.08 (0, 8.7) |
| Exome burden (N=1,261/3,420)Damaging or disruptive ultra-rare variants | 3 (2, 3.6) | 3 (2, 3.6) |

#### Data are ^1^mean (SD) or median (interquartile range). N (died/did not die) for genetic scores, CNV, exome burden.

#### **Supplementary Table 5:** Cox Proportional Hazards modelling of rare somatic mutation measures in association with all-cause mortality in Swedish cases of schizophrenia in the genetic subsample (N=4,681).

|  | **Died****(n = 1,253)** | **Did not die****(n = 3,428)** | **Hazard ratio (95%CI), p-value** | |
| --- | --- | --- | --- | --- |
|  |  |  | **Unadjusted** | **Adjusted** |
| Clonal hematopoiesis with candidate drivers | 60 (4.7%) | 69 (2.0%) | \| **2.07 (1.60-2.69), p=4.2x10^-8^** \| \| --- \| | 1.53 (1.18-1.99), p=0.0015 |
| Clonal hematopoiesis with unknown driver | 35 (2.7%) | 31 (0.9%) | **2.55 (1.83-3.58), p=8.9x10^-8^** | **1.77 (1.26-2.49), p=0.0009** |

Hazard Ratios (95% confidence interval) are presented. Bolding indicates effect was statistically significant at p≤0.0011. Adjusted model includes the exposure, and age, sex, and first 5 ancestry principle components.

#### **Supplementary Table 6:** Cox Proportional Hazards modelling of rare mutation measures in association with specific causes of death in Swedish cases of schizophrenia in the genetic subsample.

|  |  |  | ***Model 1:***  ***Copy number variant (CNV) burden*** | | ***Model 2:***  ***Rare variant burden*** |
| --- | --- | --- | --- | --- | --- |
| ***Specific Cause of Death*** | ***Died from specific cause, n*** | ***Died from other cause, n*** | ***CNV size*** | ***Number of CNVs*** | ***ddURVs*** |
| Any cancer | 205/230 | 940/1,031 | 0.96 (0.91-1.01) | 1.09 (0.93-1.26) | 1.01 (0.90-1.14) |
| Breast cancer | 11/11 | 1,134/1,250 | 0.80 (0.61-1.05) | 1.57 (0.69-3.56) | 1.34 (0.75-2.38) |
| Lung cancer | 57/67 | 1,088/1,194 | 0.99 (0.92-1.04) | 0.92 (0.69-1.24) | 1.10 (0.88-1.38) |
| Cardiovascular disease | 290/325 | 855/936 | 0.98 (0.94-1.03) | 1.02 (0.90-1.16) | 0.94 (0.86-1.04) |
| Stroke | 56/59 | 1,089/1,202 | 1.02 (0.94-1.12) | 0.80 (0.58-1.09) | 1.14 (0.90-1.45) |
| External causes | 70/76 | 1,075/1,185 | 1.00 (0.92-1.09) | 1.07 (0.84-1.36) | 1.04 (0.84-1.28) |
| Suicide | 28/30 | 1,117/1,231 | 1.03 (0.90-1.18) | 1.05 (0.73-1.50) | 0.75 (0.55-1.03) |
| Other causes | 364/395 | 781/866 | 0.99 (0.95-1.03) | 0.95 (0.85-1.07) | 1.05 (0.96-1.15) |

Hazard Ratios (95% confidence interval) are presented. No effects were statistically significant at *p*≤0.0011. N are represented as CNV / Exome burden measures. Total N for CNV (n=1,145 SCZ cases who died) and exome burden (n=1,261 SCZ cases who died). Both models contain the listed exposure (Model 1: CNV size and total number of CNVs and Model 2: ddURVs) and the first 5 ancestry principal components. Model 2 additionally contains the count of synonymous variants as a technical covariate. CNV: Copy number variant, ddURV: damaging or disruptive ultra-rare variant.

#### **Supplementary Table 7:** Cox Proportional Hazards modelling of clonal hematopoiesis somatic mutations in association with specific causes of death in Swedish cases of schizophrenia (genetic subsample, n=1,115).

| **Specific cause of death** | **Clonal hematopoiesis with candidate drivers** | | **Clonal hematopoiesis with unknown drivers** | |  |
| --- | --- | --- | --- | --- | --- |
|  | **Unadjusted** | **Adjusted** | **Unadjusted** | **Adjusted** | |
| Any cancer | 1.20 (0.67-2.15) | 1.30 (0.72-2.34) | \| 1.14 (0.51-2.57) \| \| --- \| | 1.34 (0.59-3.05) | |
| Lung cancer | 1.04 (0.33-3.31) | 1.19 (0.37-3.82) | \| 0.66 (0.09-4.75) \| \| --- \| | 0.84 (0.12-6.15) | |
| Cardiovascular disease | \| 0.68 (0.36-1.27) \| \| --- \| | 0.72 (0.38-1.36) | \| 1.04 (0.52-2.10) \| \| --- \| | 1.11 (0.55-2.25) | |
| Stroke | \| 2.71 (1.23-5.96) \| \| --- \| | 2.24 (1.01-4.98) | \| 2.84 (1.03-7.85) \| \| --- \| | 2.00 (0.71-5.60) | |
| Other causes | 1.23 (0.79-1.92) | 1.24 (0.80-1.94) | \| 1.61 (0.94-2.74) \| \| --- \| | 1.66 (0.97-2.84) | |

Hazard Ratios (95% confidence interval) are presented. No effects were statistically significant at *p*≤0.0011. Unadjusted model includes only the specified exposure. Adjusted model includes the exposure, and age, sex, and first 5 ancestry principle components. Deaths from either breast cancer or external causes did not have either measure of somatic mutations.
