# Supplementary figures and images for: The association between family history and genomic burden with schizophrenia mortality: a Swedish population-based register and genetic sample study
Source: Transl Psychiatry. 2021 Mar 15;11:163. doi: 10.1038/s41398-021-01282-1 (PMC7960991; doi:10.1038/s41398-021-01282-1)

# Supplementary Figure 1

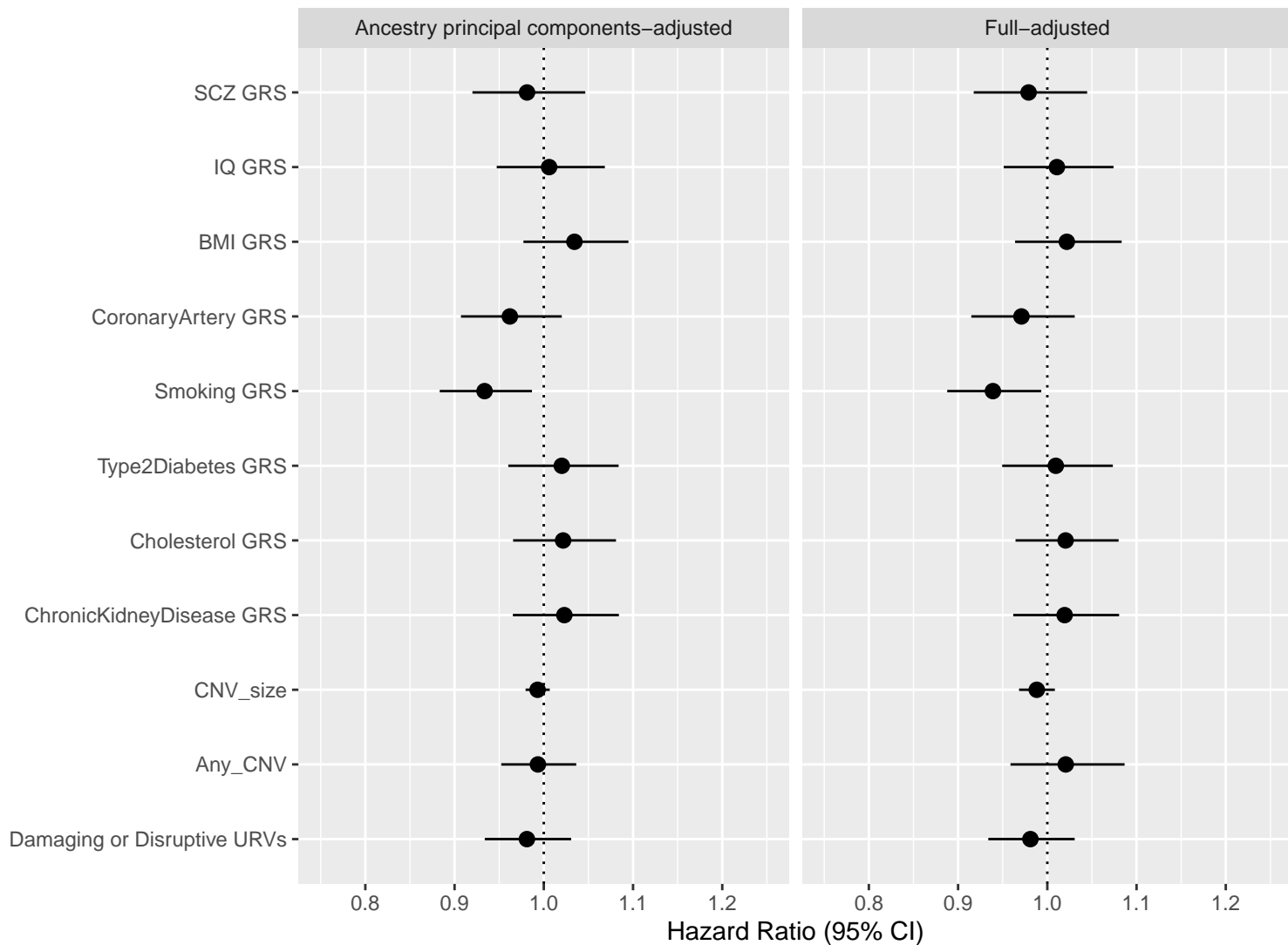

Supplement: Supplementary file 2 — Figure S1 [file 41398_2021_1282_MOESM2_ESM.pdf]
